# Baseline terminal ileal CT and MRI measurements are associated with imaging outcomes in pediatric Crohn’s disease: a cohort study

**DOI:** 10.1007/s00247-025-06302-6

**Published:** 2025-07-03

**Authors:** Allison D. Ta, Jonathan R. Dillman, Nicholas J. Ollberding, Yael Haberman, Robert Baldassano, James Markowitz, Anthony Otley, Jennifer L. Dotson, Marian Pfefferkorn, Jeffrey S. Hyams, Melvin B. Heyman, Sandra C. Kim, Joshua Noe, Scott Snapper, Adina Alazraki, Subra Kugathasan, Lee A. Denson

**Affiliations:** 1 Department of Gastroenterology, Cincinnati Children’s Medical Hospital Center, 3333 Burnett Ave, MLC 2010, Cincinnati, OH 45229 USA; 2 University of Cincinnati College of Medicine, Cincinnati, OH USA; 3 Department of Radiology, Cincinnati Children’s Medical Hospital Center, Cincinnati, OH USA; 4 Division of Biostatistics and Epidemiology, Cincinnati Children’s Medical Hospital Center, Cincinnati, OH USA; 5 Department of Gastroenterology, Sheba Medical Center, affiliated with the Tel-Aviv University, Tel-Hashomer, Israel; 6 Department of Gastroenterology, The Children’s Hospital of Philadelphia, Philadelphia, PA USA; 7 Department of Gastroenterology, Cohen Children’s Medical Center of New York, New Hyde Park, NY USA; 8 Department of Pediatrics, IWK Health Centre, Halifax, NS Canada; 9 Department of Gastroenterology, Nationwide Children’s Hospital, Columbus, OH USA; 10 Department of Gastroenterology, Indiana University School of Medicine, Indianapolis, IN USA; 11 Department of Gastroenterology, Connecticut Children’s Medical Center, Hartford, CT USA; 12 Department of Gastroenterology, University of California San Francisco, San Francisco, CA USA; 13 Department of Gastroenterology, Cleveland Clinic Children’s, Cleveland, OH USA; 14 Department of Gastroenterology, Medical College of Wisconsin, Milwaukee, WI USA; 15 Department of Gastroenterology, Boston Children’s Hospital, Boston, MA USA; 16 Department of Radiology, Emory University and Children’s Healthcare of Atlanta, Atlanta, GA USA

**Keywords:** Child, Computed tomography, Crohn’s disease, Luminal narrowing, Magnetic resonance enterography, Pediatric, Quantitative imaging, Stricture, Treatment response

## Abstract

**Background:**

Cross-sectional imaging is increasingly used for both initial diagnosis and long-term monitoring of Crohn’s disease. The quantitative morphology of the terminal ileum may predict treatment response.

**Objective:**

We aimed to identify baseline qualitative and quantitative imaging features that are associated with clinical and radiologic treatment response in a large cohort of children with Crohn’s disease.

**Materials and methods:**

This was a retrospective study of the RISK cohort study in pediatric Crohn’s disease. This multicenter study included 1,136 children <18 years from 28 sites in North America. Subjects enrolled with newly diagnosed Crohn’s disease who underwent endoscopy with baseline and follow-up CT or MRI were considered for this study. Exclusion criteria were incomplete data or surgical resection prior to follow-up imaging. Imaging analysis included assessing a qualitative terminal ileum (TI) categorical score based on SAR-AGA consensus definitions ((1) normal, (2) inflammation only without luminal narrowing, (3) inflammation with luminal narrowing, or (4) stricture with pre-stenotic dilation ≥3 cm) and quantitative measurements (maximum bowel wall thickness and maximum/minimum lumen diameter). Two endpoints were considered: (1) clinical response (off corticosteroids and quiescent Physician Global Assessment at follow-up imaging) and (2) CT and MRI response (follow-up imaging normalization). Multivariable logistic regression analyses were developed for each endpoint.

**Results:**

Ninety-six subjects were included. Clinical response endpoint was achieved in 38% (*n*=36) of participants, and imaging normalization was achieved in only 20% (*n*=19) of participants. Follow-up imaging showed disease progression in 24 (25%) patients, 7 (7%) of whom were radiologically normal at baseline (7%). A higher baseline TI categorical score was associated with lower odds of imaging normalization during follow-up (OR 0.4 [0.2, 0.8], *P*=0.009). Larger TI minimum lumen diameter (OR 1.1 [1.01, 1.3], *P*=0.04) and smaller maximum bowel wall thickness at baseline (OR 0.8 [0.6, 0.97], *P*=0.03) were associated with imaging normalization. There were no baseline imaging measurements associated with clinical response.

**Conclusions:**

Baseline increased terminal ileal minimum lumen diameter and decreasing wall thickness were associated with imaging normalization at follow-up, but not clinical response.

**Graphical Abstract:**

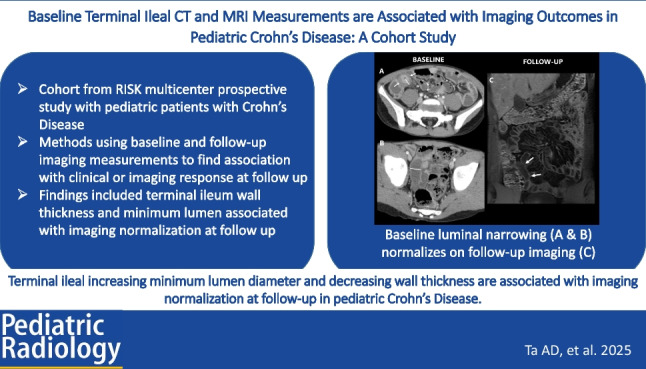

**Supplementary Information:**

The online version contains supplementary material available at 10.1007/s00247-025-06302-6.

## Introduction

Crohn’s disease (CD) is an autoimmune disease with transmural tissue inflammation along the gastrointestinal tract. Inflammatory disease can progress to more complicated disease with smooth muscle proliferation and fibrostenosis [1, 2]. Endoscopic evaluation is the gold standard for diagnosis; however, cross-sectional imaging continues to be important for assessing baseline disease and disease monitoring. Despite modern medications and therapeutic drug monitoring, healing still only occurs in a minority of patients. With rising pediatric disease incidence and complications developing in 7–25% of patients following diagnosis, imaging can play a key role in identifying pediatric patients at risk for these complications [3–5].

**Fig. 1:**
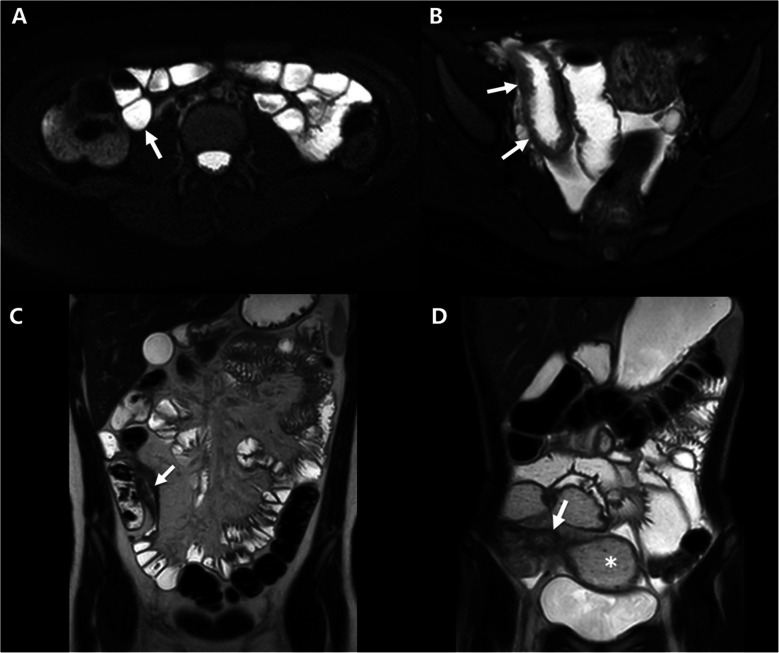
Examples of MRI images of the AGA-SAR classification system. **a** A 13-year-old with Crohn’s disease. Axial T2-weighted single-shot fast spin-echo image of the terminal ileum (*arrow*) appears normal without wall thickening or luminal narrowing. **b** A 16-year-old with Crohn’s disease. Axial T2-weighted single-shot fast spin-echo image shows terminal ileal wall thickening (*arrows*), but no luminal narrowing. There is also intramural edema and mucosal ulcers. **c** A 16-year-old with Crohn’s disease. Coronal T2-weighted single-shot fast spin-echo image shows terminal ileal wall thickening and luminal narrowing (*arrow*), but no dilation of the upstream bowel. **d** A 14-year-old with Crohn’s disease. Coronal T2-weighted single-shot fast spin-echo image shows terminal ileal wall thickening and luminal narrowing (*arrow*) as well as upstream dilation of the more proximal ileum and the small bowel feces sign (*asterisk*)

Cross-sectional imaging, including computed tomography (CT) and magnetic resonance imaging enterography (MRE), is used as a non-invasive method to evaluate the presence, location, and severity of inflamed bowel segments as well as strictures and possible co-existing penetrating complications in pediatric Crohn’s Disease [6]. Historically, a lack of standard definitions for luminal narrowing and stricture has made interpretation of results challenging across studies [7, 8]. In the 2018 American Gastroenterological Association (AGA)-Society of Abdominal Radiology (SAR) consensus guidelines, which were endorsed by the Society for Pediatric Radiology, the nomenclature for luminal narrowing and stricture was strictly defined. Luminal narrowing occurs without pre-stenotic dilation, while a stricture is a segment of intestine showing wall thickening, luminal narrowing >50%, and pre-stenotic dilation ≥3 cm (Fig. 1) [9]. Incorporating the 2018 definitions has led to improved predictive models of transmural healing in pediatric patients [10].

Quantitative imaging assessments of bowel wall inflammation using MRE have been developed into scoring systems, including the MR index of activity (MaRIA), simplified MaRIA, and London score [11–13]. These scores include both qualitative measurements (such as edema, ulceration, or fat stranding) and quantitative measurement of bowel wall thickness. The MaRIA score uses wall thickness as a continuous variable, while other scores use dichotomous or categorical assessment of the bowel wall. Imaging scoring systems have been used extensively in research studies, but clinically, their use can be limited by the time required for scoring, and/or the variation of inflammation along bowel segments may not be captured in these scores [9, 12–14].

We aimed to perform both qualitative (based on the AGA-SAR guidelines) and quantitative assessments (minimal lumen diameter, maximal lumen diameter, and bowel wall thickness) of terminal ileal morphology at diagnosis and evaluate their associations with eventual clinical response and imaging normalization (i.e., radiologic transmural healing) at follow-up. Secondly, we aimed to develop a multivariable logistic regression model to assess the association between qualitative and quantitative radiologic assessments of the terminal ileum at diagnosis with eventual clinical response and imaging normalization (i.e., radiologic transmural healing) at follow-up.

## Methods

### Study design

This study analyzed data from a sub-cohort of the RISK cohort study which enrolled 1,136 children at the time of diagnosis with Crohn’s disease from 2008–2012 at 28 sites across North America and followed them for a total of 5 years (ClinicalTrials.gov identifier NCT00790543) [3].

### Ethical considerations

The institutional review board at each site reviewed the RISK study protocol prior to approval, which allows for additional analysis of completed data. Appropriate written informed consent was obtained in all cases from parents/guardians or patients, and informed assent was provided as appropriate.

**Fig. 2:**
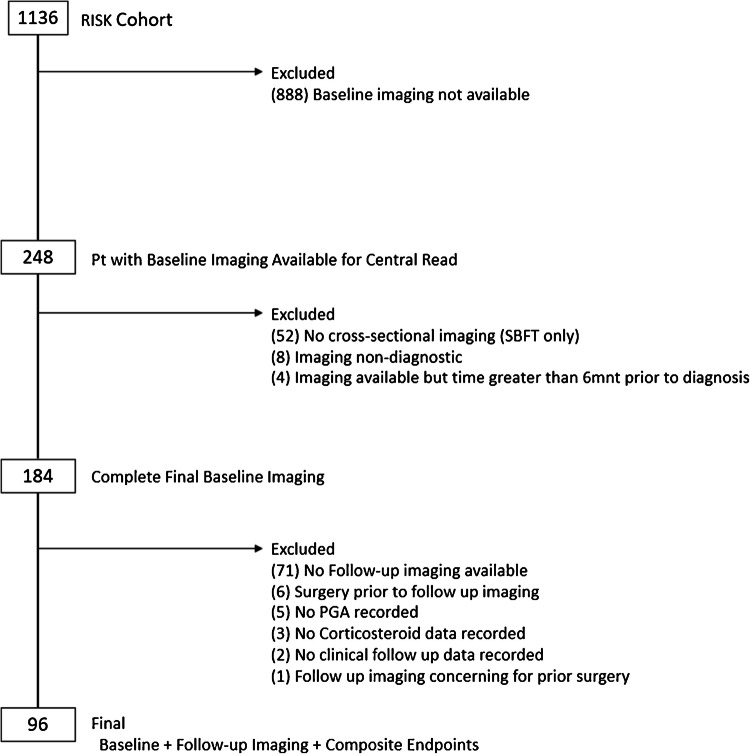
Consort diagram for study population

Subjects who had suspected Crohn’s disease and underwent baseline endoscopy to confirm disease were enrolled. Baseline clinical, demographic, serologic, imaging, and endoscopic data were collected. Inclusion criteria for the current study were enrollment in RISK and complete clinical/demographic information. Expanding beyond prior publications of the RISK cohort, inclusion criteria for imaging were more stringent by including only diagnostic cross-sectional imaging (CT and MRI) to facilitate quantitative measurements on baseline imaging (within 6 months of diagnosis) and follow-up imaging (6 months after diagnosis) [3, 10]. The current analysis, therefore included 71 out of 96 RISK cohort study participants included in a prior publication from our group, which focused upon associations between baseline qualitative ileal features and concurrent gene expression and microbial community features [12]. An additional 25 subjects from the RISK cohort with baseline and follow-up imaging were also included. Participants were excluded based on incomplete data or surgical resection prior to follow-up imaging (Fig. 2).

Baseline data were obtained, including demographics, clinical history, antimicrobial serology, and cross-sectional imaging (CT or MRI). Prior to treatment, a baseline ileocolonoscopy was performed to confirm histologic disease characteristics. Montreal classification was used for disease behavior (B1-non-stricturing, non-penetrating; B2-structuring; or B3-penetrating) [15]. As part of RISK, every 6 months after diagnosis, all subjects were evaluated regarding current medications (including biologic therapy and corticosteroid use), anthropometric measurements, documentation of interval imaging, and surgery/hospitalization since the prior visit. Medical management was based upon the patient’s individual physician’s assessment and recommendations. For the purposes of this study, data collected from the RISK database included age at enrollment, sex, Physician Global Assessment status at baseline and follow-up, use of corticosteroid, use of anti-TNF-α therapy within 3 months of diagnosis, and use of anti-TNF-α therapy within 3 years of diagnosis.

The co-primary outcomes were evaluated at the time of follow-up imaging as: (1) clinical response or (2) imaging normalization response. For the clinical response, this was defined as corticosteroid-free and Physician Global Assessment (PGA) quiescent, at clinical follow-up coinciding with follow-up imaging. The PGA is a clinical score determined by the subject’s medical provider, ranging from quiescent, mild, moderate, to severe disease activity [16]. For imaging normalization response, this was defined as radiologic transmural healing without active inflammation, no bowel wall thickening, or luminal narrowing on follow-up cross-sectional imaging (CT or MRI) based on central imaging review.

### Imaging analysis

Centralized reading was performed for all relevant imaging by a board-certified fellowship-trained pediatric radiologist with 13 years post-fellowship clinical experience. Cross-sectional imaging included clinical abdominopelvic computed tomography (CT) with intravenous contrast material, computed tomography enterography (CTE), magnetic resonance imaging (MRI), and magnetic resonance enterography (MRE) (Supplemental Table 1). Imaging was performed based on local institutional protocols. As this study utilized clinical imaging exams, there were slight differences between imaging studies with respect to pulse sequences obtained and imaging planes. Baseline imaging examinations obtained within 6 months of diagnosis were analyzed, while imaging obtained more than 6 months after diagnosis was defined as follow-up.

### Qualitative analysis

Ileal CD involvement was qualitatively assessed (i.e., TI categorical score) by the AGA-SAR consensus guidelines as follows: (1) no active inflammation; (2) active inflammation only (abnormal post-contrast hyperenhancement and wall thickening) without luminal narrowing; (3) active inflammation with luminal narrowing (ileal lumen diameter reduced to ≤ 50% of the upstream small bowel luminal diameter); and (4) overt stricture defined as wall thickening, luminal narrowing with ≥3 cm pre-stenotic bowel dilation [9, 10].

### Quantitative analysis

**Fig. 3:**
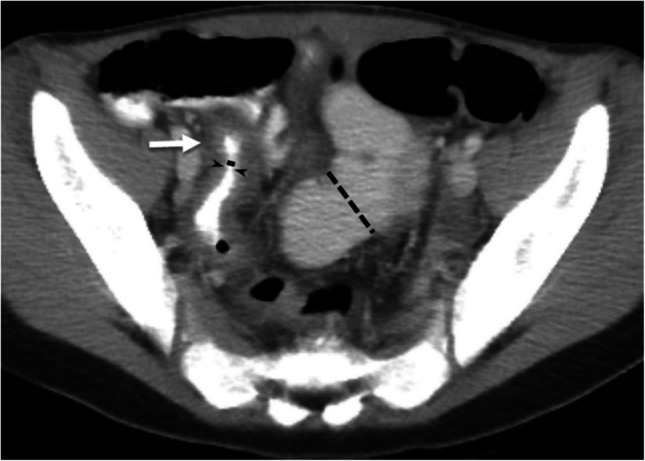
Example of terminal ileum measurements in a patient with luminal narrowing. A 13-year-old with Crohn’s disease. Axial CT image with IV and oral contrast material which demonstrates luminal narrowing and wall thickening of the terminal ileum. The minimum luminal diameter was approximately 1 mm (*solid black line*) while there was proximal terminal ileum maximal dilation of 28 mm (*black dash line*) which generated a luminal narrowing ratio of 28. The maximal wall thickness was 7 mm measured at the level of the *white arrow*

Quantitative measurements were made on the imaging series (sequence, plane) that best demonstrated the imaging feature/measurement of interest. Both non-contrast and post-contrast images were used for this study and varied by patient. Measurements were within the terminal ileum lumen, defined as the 15 cm of ileum proximal to and including the ileocecal valve (Fig. 3). Maximal wall thickness was measured in the thickest area from the inner wall (mucosa, if present) to the outer wall (serosa), then perpendicular to the luminal centerline. The minimal lumen diameter was measured at the area with the smallest diameter from the inner wall to the inner wall, orthogonally to the luminal centerline. The maximal lumen diameter was measured at the area with the largest diameter from the inner wall to the inner wall, orthogonally to the luminal centerline. A luminal narrowing ratio of maximal to minimal luminal diameter was calculated to create an objective luminal narrowing “index” where no luminal narrowing =1 and increasingly positive values are indicative of worsening stricturing disease (i.e., more severe luminal narrowing and greater proximal bowel dilation).

### Statistical analysis

Continuous variables were summarized as medians and interquartile ranges, while categorical variables were summarized as counts and percentages. Fisher’s exact or chi-squared and Wilcoxon rank-sum tests were used to test differences in categorical and continuous variables, respectively, between patient subgroups. Wilcoxon signed-rank test was used to test for differences in paired data. Multivariable logistic regression was used to obtain covariate-adjusted odds ratios (OR) and 95% confidence intervals (CIs) for achievement of each co-primary endpoint according to baseline imaging factors. All non-imaging model covariates were determined a priori and included age at diagnosis, sex, race (Caucasian vs. other), and duration of follow-up. All analyses were performed using the R program for statistical computing and graphics (R Foundation for Statistical Computing, Vienna, Austria, 2022) [17]. *P*-values <0.05 were considered significant for inferential testing.

### Data availability

All data supporting the findings of this study are available from the corresponding author upon reasonable request.

## Results

Of the 1,136 patients in the RISK cohort, a total of 96 were included in this study based on the availability of baseline and follow-up imaging for central reading (Table 1). Imaging response was achieved in 20% (*n*=19) while 80% were imaging non-responders. Clinical response outcome, defined as quiescent PGA and corticosteroid-free at the time of follow-up, was achieved by 38% (*n*=36) of subjects, while clinical non-responders were 62% of the total study cohort (Table 2).

**Table 1:** Demographics and baseline characteristics of clinical and imaging non-responders and responders

	Total study cohort	Clinical		Imaging normalization	
		Non-responders	Responders	Non-responders	Responders
	*N*=96	*N*=60	*N*=36	*N*=77	*N*=19
**Baseline demographics**					
Age at diagnosis (years)	11.9 (10, 14.4)	11.9 (10, 14.4)	11.6 (10.1, 14)	11.8 (10.2, 13.8)	12.4 (10, 15)
Male	60 (62%)	37 (62%)	23 (64%)	52 (68%)	8 (42%)
Caucasian	79 (82%)	49 (82%)	30 (83%)	62 (81%)	17 (89%)
Physician Global Assessment moderate-severe	63 (66%)	41 (68%)	22 (61%)	48 (62%)	15 (79%)
Time to baseline imaging (month)	0.2 (−0.07, 0.7)	0.2 (−0.1, 0.7)	0.3 (0.03, 0.9)	0.2 (−0.03, 0.8)	0.4 (−0.1, 0.6)

Clinical response was based on quiescent PGA and off corticosteroids at time of follow-up

Data presented as median (25% IQ, 75% IQ) or number (frequency in cohort)

**Table 2:** Baseline and follow-up imaging characteristics of clinical and imaging responders

	Total study cohort	Clinical		Imaging normalization	
		Non-responders	Responders	Non-responders	Responders
	*N*=96	*N*=60	*N*=36	*N*= 77	*N*=19
**Baseline TI radiologic categorization**					
Normal (1)	13 (14%)	8 (13%)	5 (14%)	7 (9%)	6 (32%)
Inflammation only (2)	31 (32%)	17 (28%)	14 (39%)	25 (32%)	6 (32%)
Luminal narrowing (3)	50 (52%)	34 (57%)	16 (44%)	43 (56%)	7 (36%)
Stricture (4)	2 (2%)	1 (2%)	1 (3%)	2 (3%)	0
**Follow-up TI radiologic categorization**					
Normal (1)	19 (20%)	8 (13%)	11 (31%)	0	19 (100%)
Inflammation only (2)	21 (22%)	13 (22%)	8 (22%)	21 (27%)	0
Luminal narrowing (3)	50 (52%)	34 (57%)	16 (44%)	50 (65%)	0
Stricture (4)	6 (6%)	5 (8%)	1 (3%)	6 (8%)	0
**Follow-up clinical status**					
Months to imaging follow-up	33 (15, 50)	25 (14, 48)	39 (20, 53)^†^	28 (15, 49)	42 (22, 54)
Months to clinical follow-up	32 (15, 49)	26 (14, 45)	37 (20, 54)^†^	26 (15, 47)	39 (22, 54)
PGA moderate-severe	25 (26%)	25 (42%)	0^‡^	21 (27%)	4 (21%)
CS ever at 3 years	84 (88%)	55 (92%)	29 (81%)	67 (87%)	17 (89%)
CS at follow-up	29 (30%)	29 (48%)	0^‡^	25 (32%)	4 (21%)
Anti-TNF within 3 months	23 (29%)	13 (25%)	10 (34%)	17 (26%)	6 (40%)
Anti-TNF within 3 years (*N* = 81)	80 (83%)	51 (85%)	29 (81%)	65 (84%)	15 (79%)

Clinical response was based on quiescent PGA and off corticosteroids at time of follow-up

*PGA*, Physician Global Assessment; *TNF*, tumor necrosis factor; *CS*, corticosteroid

Data presented as median (25% IQ, 75% IQ) or number (frequency in cohort)

^†^*P*-value <0.05, ^‡^*P*-value <0.001

Baseline demographics and serology were similar between non-responder and responder groups for each primary outcome (Tables 1 and 2). Anti-TNF-α therapy was used within 3 months in 23 subjects and increased to 80 subjects by the three-year follow-up. There were no statistically significant differences in the use of anti-TNF-α treatment between clinical responders and non-responders in the first 3 months from diagnosis or at three-year RISK follow-up. Early anti-TNF-α therapy was used in 26% (*n*=17) of imaging non-responders and 40% (*n*=6) of imaging responders; however, this difference was not statistically significant. For the imaging normalization endpoint, patients whose follow-up imaging demonstrated non-response were noted to have their follow-up imaging earlier than those whose follow-up imaging demonstrated response, though not by a statistically significant difference (median 28 months [15, 49] vs. 42 [22, 54] (*P*=0.07)). Within the clinical non-response vs. response subgroups, follow-up imaging was obtained sooner in the non-response subgroup (median of 25 months [14, 48] vs. 39 [20, 53] (*P*=0.02)).

### Qualitative TI radiologic categorization

Ileal categorization remained stable between baseline and follow-up in 55% (*n*=53) of subjects (Table 3). Improvement from baseline to follow-up was seen in 6 subjects with inflammation at baseline and 13 with luminal narrowing at baseline. However, 25% (*n*=24) of patients had progression of their ileal categorization. The two patients with strictures at baseline had persistent strictures on follow-up imaging. There were 13 subjects with normal small bowel imaging at baseline; however, only 6 of these patients remained normal at follow-up, while the remaining progressed to inflammation (*n*=4) to luminal narrowing (*n*=2), or developed an overt stricture (*n*=1).

**Table 3:** Terminal ileum radiologic categorization at baseline and at imaging follow-up based on four AGA-SAR categories: normal (1), active inflammation without luminal narrowing (2), active inflammation with luminal narrowing (3), and stricture (4)

		Follow-up imaging				
	Categorization	Normal	Inflammatory	Luminal narrowing	Stricture	Total
**Baseline imaging**	Normal	6	4	2	1	13
	Inflammatory	6	11	14	0	31
	Luminal narrowing	7	6	34	3	50
	Stricture	0	0	0	2	2
	Total	19	21	50	6	96

### Quantitative TI measurements

Quantitative measurements of the TI lumen diameter and TI wall thickness were obtained within 15 cm of the ileocecal valve (Table 4). At baseline, there were no statistical differences in quantitative measurements between clinical responders vs. non-clinical responders or imaging responders vs. non-imaging responders. Clinical responders did have a statistically significant decrease in the TI wall thickness at follow-up compared to clinical non-responders, but no other differences were found in comparisons between clinical responders vs. clinical non-responders. At follow-up, imaging responders had an increase in the minimal lumen diameters, an increase in maximal lumen diameter, improved luminal narrowing (max/min) ratio, and decreased wall thickness compared to imaging non-responders.

**Table 4:** Terminal ileal lumen and wall thickness quantitative measurements at baseline and follow-up, stratified by clinical or imaging response

	Lumen min (mm)		Lumen max (mm)		Luminal narrowing (max/min) ratio		Wall thickness (mm)	
	Baseline	Follow-up	Baseline	Follow-up	Baseline	Follow-up	Baseline	Follow-up
**Total study cohort**	3 (1, 9)	2 (1, 10)	17 (13, 20)	17 (14, 20)	6 (2, 19)	7 (2, 18)	6 (4, 8)	6 (3, 8)
**Clinical non-responders**	3 (1, 9)	2 (1, 10)	17 (14, 21)	18 (15, 22)	6 (2, 19)	10 (2, 18)	6 (4, 8)	7 (3, 8)
**Clinical responders**	4 (1, 8)	5 (1, 9)	17 (13, 19)	16 (13, 19)	4 (2, 16)	3 (2, 12)	6 (5, 8)	5 (3, 7)^†^
**Imaging non-responders**	2 (1, 8)	1 (1, 6)	17 (13, 21)	18 (15, 22)*	6 (2, 19)	11 (3, 19)	7 (5, 8)	7 (5, 8)
**Imaging responders**	8 (1, 10)	12 (8, 13)*^‡^	17 (14, 18)	16 (14, 18)^‡^	2 (2, 11)	1 (1, 2)*^‡^	5 (2, 7)	2 (2, 3)*^‡^

Measurements made in the terminal ileum within 15 cm of the ileocecal valve. Lumen min was the minimal lumen diameter. Lumen max was the maximal lumen diameter. Data presented as median (25% IQ, 75% IQ)

*Statistical difference between baseline and follow-up (*P*<0.05)

^†^Statistical difference between clinical responders and non-responders at baseline or follow-up (*P*<0.05)

^‡^Statistical difference between imaging normalization responders and non-responders at baseline or follow-up (*P*<0.05)

### Predictors of imaging normalization endpoint

From the total study cohort, 20% (*n*=19) of subjects achieved imaging normalization (i.e., radiologic transmural healing) at follow-up imaging (Table 2). A multivariable logistic regression model was developed after adjusting for age at diagnosis, gender, Caucasian race, and duration until follow-up imaging (Table 5). Lower odds of imaging normalization with radiologic transmural healing on follow-up imaging were independently associated with a higher baseline TI categorical score (i.e., one unit increase was associated with OR =0.4 [0.2, 0.8], *P*=0.0093), greater TI wall thickness (OR=0.8 [0.64, 0.97], *P*=0.03), and smaller minimal lumen diameter (OR=1.1 [1.01, 1.3], *P*=0.04).

**Table 5:** Multivariable analysis of baseline imaging parameters

	Adjusted OR	*P*-value
**Clinical response**		
Baseline categorization (1, 2, 3, 4)	0.8 (0.5, 1.5)	0.50
Baseline lumen min	1.01 (0.9, 1.1)	0.78
Baseline lumen max	1 (0.9, 1.1)	0.97
Baseline luminal narrowing ratio (max/min)	1 (0.96, 1.1)	0.98
Baseline wall thickness	0.97 (0.8, 1.1)	0.67
**Imaging normalization**		
Baseline categorization (1, 2, 3, 4)	0.4 (0.2, 0.8)	**0.0093**
Baseline lumen min	1.1 (1.01, 1.3)	**0.04**
Baseline lumen max	0.9 (0.8, 1.1)	0.33
Baseline luminal narrowing ratio (max/min)	0.9 (0.9, 1)	0.058
Baseline wall thickness	0.8 (0.6, 0.97)	**0.03**

Multivariable analysis was conducted to test for associations between baseline imaging features and either clinical response or imaging normalization. The analysis adjusted for age at diagnosis, gender, Caucasian race, and duration of follow-up. The endpoints included: clinical response (off corticosteroids and quiescent PGA at time of follow-up imaging) and image normalization

### Predictors of clinical response endpoint

Of the 36 subjects who reached the clinical response endpoint, follow-up imaging was normal for only 31% (*n*=11) of subjects. Clinical response was not associated with any of the qualitative or quantitative baseline imaging features assessed (Table 5). Combined clinical and imaging response endpoints were achieved in only 11% (*n*=11) of all subjects.

## Discussion

This study highlights the importance of follow-up imaging despite clinical remission. While clinical improvement only occurred in 38% of this study, only 11% of the study cohort were able to achieve both clinical remission and imaging normalization. We found a significant association between baseline TI minimal lumen diameter and wall thickness with improved odds of imaging normalization with transmural healing at follow-up. Currently, there are no guidelines recommending specific timing of when follow-up imaging should be performed. In our study, follow-up imaging was a median of 33 months from diagnosis, and there was progression of disease in 24% of this cohort from their baseline categorization. Patients with stricture or penetrating CD disease were likely to have follow-up imaged sooner compared to asymptomatic patients. We also saw a discordance between patients in clinical remission and persistence of active imaging findings, which suggests that imaging identifies some subclinical inflammatory disease [18].

Cross-sectional imaging (CT and MRI) has been used as part of diagnosis and surveillance in IBD, particularly to evaluate small bowel inflammation and associated complications [19–21]. In clinical practice, cross-sectional imaging is frequently ordered to re-evaluate disease in symptomatic patients with CD. In 2018, the AGA-SAR provided new consensus guidance recommending differentiation of non-stricturing, non-penetrating inflammatory disease from luminal narrowing without proximal dilation [9]. Imaging characteristics predictive of treatment response have included severe TI disease, creeping fat, lower MaRIA score, and lower bowel wall thickness [22]. Our study supported these findings of bowel wall thickness associated with improved outcomes. Other quantitative measures of minimum and maximum lumen diameter have been studied as well. Debnath et al. performed a retrospective study in CD evaluating cross-sectional imaging predictive of surgery and found that ileal lumen max/min ratio and simplified MaRIA were associated with the need for surgery [23]. Orscheln et al. found penetrating disease was associated with small lumen diameter and/or stricture [24]. We showed comparable findings with imaging normalization (i.e., transmural healing) associated with larger minimal lumen diameter and trending towards significance for the lower max/min ratio. A larger prospective imaging study could help further elucidate the utility of these quantitative measures in the pediatric population.

Bowel segments affected by luminal narrowing and stricturing can have both inflammatory and fibrotic components as well as substantial smooth muscle proliferation, which may explain the varied response to anti-inflammatory medications [7]. Changes in gene expression towards pro-fibrotic signatures at diagnosis have been associated with luminal narrowing on imaging, which suggests luminal narrowing may be a surrogate to represent early gene expression shifts [10, 25]. While medical therapies, such as anti-TNF-α biologics, can reduce inflammation, they are not targeted at reducing fibrostenosis that can develop over time, causing bowel wall thickening and strictures. A lower risk of stricture complications has been reported with early anti-TNF-α exposure [26]. In our study, imaging responders (radiologic transmural healing) had increased early anti-TNF-α exposure, but this was not statistically significant; this may be because a longer duration of therapeutic biologic drug exposure may be necessary to achieve this goal, but is not feasible in an observational study.

The strengths of this study include the use of a large sub-cohort of the RISK study, assessment of the recently described AGA-SAR categorization system, and the use of both clinical and transmural healing as endpoints. Also, this study included quantitative measurements of the TI to correlate with key outcomes. Limitations include the lack of systematic follow-up imaging at regular intervals and the heterogeneous nature of clinical CT and MRI. Unlike a randomized controlled trial, the RISK cohort study was an observational study, and, thus, there were no systematic treatment protocols and therapeutic drug monitoring (i.e., patients were managed based on institutional standard of care practices), making it difficult to assess the impact of medical therapies on the outcomes. Future studies where therapy protocols are regimented with regular imaging could further evaluate this question.

In conclusion, TI qualitative and quantitative measurements from baseline cross-sectional CT and MRI small bowel imaging are associated with imaging normalization at follow-up in pediatric CD. Qualitative categorization based on the AGA-SAR guidelines was the strongest predictor of imaging response, and multiple quantitative measurements were predictors of imaging normalization in this study. There were no associations between baseline imaging features and clinical response. Findings of luminal narrowing may become modifiable therapeutic targets for both current anti-inflammatory medications as well as future clinical trials for anti-fibrotic therapies. Future prospective studies are needed to further understand how baseline imaging can be used to direct individual patient care and ultimately improve clinical outcomes.

## Supplementary Information

Below is the link to the electronic supplementary material.

Supplementary file1 (DOCX 31 KB)

Supplementary file2 (DOCX 15 KB)
