# Supplementary material for: Baseline terminal ileal CT and MRI measurements are associated with imaging outcomes in pediatric Crohn’s disease: a cohort study
Source: Pediatr Radiol. 2025 Jul 3;55(8):1642–51. doi: 10.1007/s00247-025-06302-6 (PMC12321652; doi:10.1007/s00247-025-06302-6)

**Supplemental Figure 1.** **Venn diagram of overlap between clinical and imaging responders.** There were a total of 36 subjects with clinical response and a total of 19 subjects with imaging response. Only 11 subjects had both clinical and imaging response.


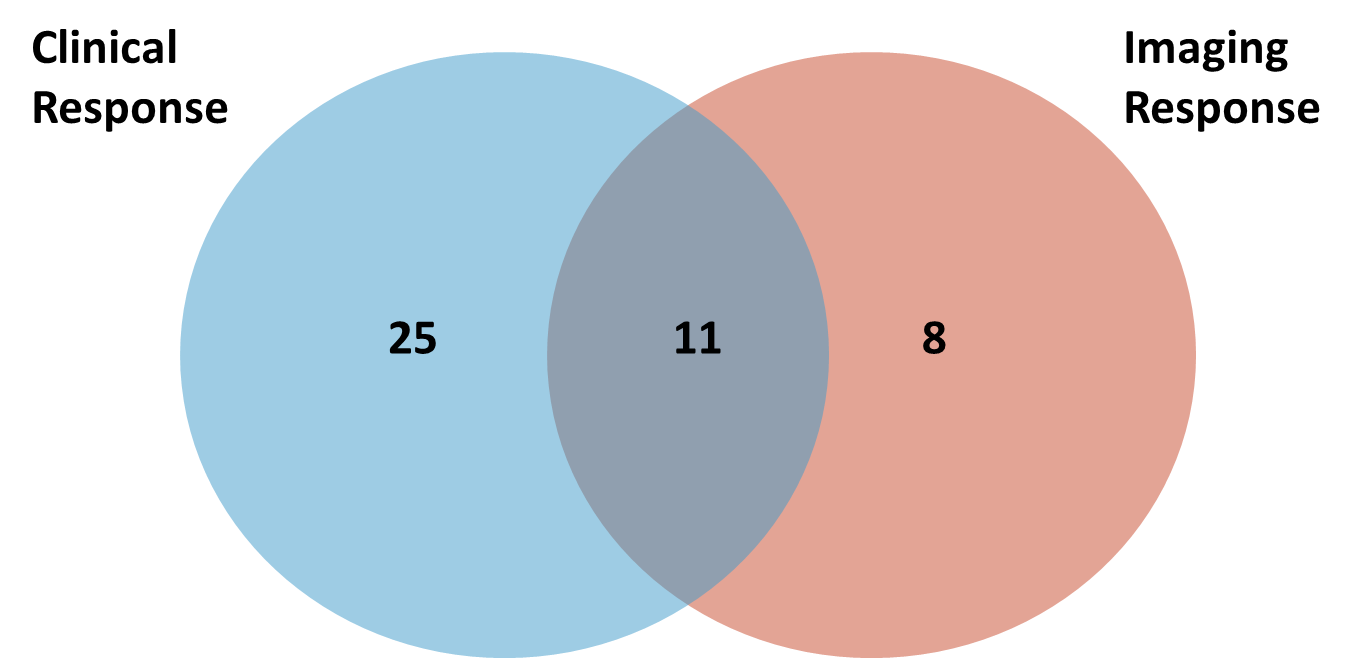

Supplement: Supplementary file 1 — Supplementary file1 (DOCX 31 KB) [file 247_2025_6302_MOESM1_ESM.docx]
