# Supplementary material for: Baseline terminal ileal CT and MRI measurements are associated with imaging outcomes in pediatric Crohn’s disease: a cohort study
Source: Pediatr Radiol. 2025 Jul 3;55(8):1642–51. doi: 10.1007/s00247-025-06302-6 (PMC12321652; doi:10.1007/s00247-025-06302-6)
Supplement: Supplementary file 2 — Supplementary file2 (DOCX 15 KB) [file 247_2025_6302_MOESM2_ESM.docx]

**Supplemental Table 1.** **Baseline and follow up imaging by type of study and achieving the study endpoints.**

|  | **Clinical Response** | | **Imaging Normalization** | |
| --- | --- | --- | --- | --- |
|  | **Non-Responders** | **Responders** | **Non-Responders** | **Responders** |
|  | 60 | 36 | 77 | 19 |
| **Baseline Imaging** |  |  |  |  |
| CT | 14 | 4 | 12 | 6 |
| CTE | 11 | 3 | 13 | 1 |
| MRE | 33 | 29 | 50 | 12 |
| MRI | 2 | 0 | 2 | 0 |
| **Follow-up Imaging** |  |  |  |  |
| CT | 2 | 1 | 3 | 0 |
| CTE | 6 | 3 | 7 | 2 |
| MRE | 52 | 32 | 67 | 17 |
